# Supplementary material for: Redundant prefrontal hemispheres adapt storage strategy to working memory demands
Source: Nat Commun. 2026 Jul 20;17:8858. doi: 10.1038/s41467-026-75705-2 (PMC13500473; doi:10.1038/s41467-026-75705-2)
Supplement: Supplementary file 3 [file 41467_2026_75705_MOESM3_ESM.pdf]

nature portfolio

Peer Review File

Redundant prefrontal hemispheres adapt storage strategy to working memory demands

Corresponding Author: Dr Albert Compte

This file contains all reviewer reports in order by version, followed by all author rebuttals in order by version.

Version 0:

Reviewer comments:

Reviewer #1

(Remarks to the Author)
Review: Redundant prefrontal hemispheres adapt storage strategy to working memory demands

Authors: Tschiersch, Umakantha, Williamson, Smith, Barbosa and Compte

Summary

In this manuscript, the authors combine neural recordings in monkeys performing a visuo-spatial working memory task with artificial neural network (ANN) modeling to investigate how working memory (WM) is distributed across hemispheres. Using bilateral recordings in lateral prefrontal cortex (lPFC, area 8Ar) and population decoding, they replicate previous findings that both hemispheres represent the location of the target during the delay, with stronger representation in the contralateral hemi-field. Additionally, they demonstrate that in both hemispheres, population decoder errors (clockwise (CW) and counterclockwise (CCW)) correlate with behavioral errors, indicating that both hemispheres could jointly support behavior. However, this error correlation was stronger within than across hemispheres, suggesting that WM representations are weakly coupled between hemispheres. This finding is corroborated by their ANN modeling, which additionally predicted that serial dependency is hemisphere-specific in their neural recordings. Finally, they show that in their two weakly coupled single-item capacity ring attractor network model, adding a second item leads to lateralization. From this alone, they conclude that storage strategy (lateralized vs. redundant) depends on cognitive demand.

Overall, this manuscript combines neural data analyses with ANN modeling to address an interesting question in the field—how spatial WM is shared across hemispheres. The ANN model makes specific predictions about error correlations and the effect of trial history within and across hemispheres that are corroborated by their neural data analyses. These results are convincing and clearly presented. My main concern is that their result on cognitive demand appears specific to their modeling choice and cannot be tested with their data. Although this is their weakest result, they rely heavily on it in their conclusion (and even the title), which diminishes my enthusiasm for the paper. Below, I expand on this concern and other aspects of the manuscript.

Major Comments

As stated above, my primary concern regards the final section (Figure 6), where the authors show that in their two weakly coupled single-item capacity ring attractor network model, adding a second item and a lateralization signal leads to: (i) contralateral memory specialization and (ii) reduced memory capacity for items presented in the same hemi-field.
I don't believe that a single-item capacity ANN is an appropriate model of WM for multiple stimuli. Since the ANN cannot hold two items within the same hemisphere, it seems trivial that when presented with two items, it will forget one and is more likely to forget the stimulus with the weaker input (from the ipsilateral hemifield). I believe a more realistic model with multiple-item capacity and account for interference would be more appropriate and will make more realistic predictions (e.g., references 37-41 and Compte et al., 2000; and see below for why a pure slot model is unlikely to be a good model for lPFC). The main weakness of these results is that the specific prediction that their model makes (e.g., one item is randomly forgotten when two items are presented in the same hemifield if the other one is redundantly represented) cannot be tested with their data, as they do not manipulate the load. Additionally, a pure slot model is unlikely to be a good model for WM in lPFC, as information about items in working memory has been shown to be reduced when more items are added in the

same hemi-field rather than being 'all or none' (Buschman et al., 2011).
Overall, this is their weakest result, and I do not believe it should feature so prominently in their interpretation. In particular, the title is misleading.

I find it difficult to reconcile the findings described in Figure 2h (greater decoding accuracy of the target location for the contralateral hemi-field) and Figure 3c (no difference in the brain-behavior correlation on a trial-by-trial basis). The authors conclude that both hemispheres are equally involved in guiding behavior. It appears that the analysis in Figure 3c is noisier, and the absence of difference between the hemi-fields represents a null effect.

Figure 3e: Error correlation is significantly above zero before stimulus onset (also in Figure S5c). Can the authors explain this? This is particularly concerning for the 'within' curve, which does not seem be above this 'baseline.'

Minor Comments

• Figure 1d is difficult to read. I recommend using a color gradient and making this panel larger (as in Wimmer et al., 2014).
• Model lines are missing from Figure 1f.
• Monkey Pe has a very low number of multi-units that are selective for a location during the delay compared to the two other monkeys (Figure 2a legend). Can the authors explain this?
• Figure 2h and lines 190/198, page 8: The authors state that memory reactivation has been previously shown to correlate with serial dependence. Can they demonstrate this in their data as well?
• Lines 202-207, page 8: It took me some time to realize that the behavioral and neural decoder errors were signed (CW/CCW). Could the authors make this clearer in the text?
• Lines 202-207, page 8: Why use the slope as your statistical test for correlation? It seems that a significant correlation is what matters (as an area under the curve if the timing is uncertain).

(Remarks on code availability)

Reviewer #2

(Remarks to the Author)
The authors combine electrophysiological recordings from macaque monkeys performing a visuospatial task and neural network simulation to elucidate two alternative mechanisms of interhemispheric coding for working memory across the entire visual space: specialized coding vs. redundant coding. In electrophysiological recordings, the authors performed simultaneous recordings on the bilateral prefrontal cortex of a monkey and found redundant coding in each hemisphere with weak coupling; in modeling, they not only replicate the main findings from the experiments but also demonstrate the switch between specialized coding and redundant coding, which depends on the task demand.

This is a very interesting and systematic work. The authors carefully select the within-trial imprecision and the across-trial serial dependence, both of which are closely related to working memory coding and operate on different time scales. Based on the experimental findings, they construct a redundant, weakly connected network with lateralized inputs, which ingeniously reconciles the two interhemispheric coding mechanisms.

I just have some points which may be helpful to improve the manuscript:
1. I noticed that in neural data, the across-area error correlation seems significantly larger than 0 before the stimulus presentation (Fig. 3e), while the same quantity is close to 0 in the model (Fig. 4e). I'm wondering how the authors interpret this discrepancy. I think explaining the non-zero across-area error correlation in neural data is important, as this is the major evidence supporting interhemispheric correlations.
2. In the model with weak across-area connectivity (Fig. 3h), the authors found that the across-hemisphere reactivation drift is much smaller than the within-hemisphere one. However, it seems that this across-hemisphere reactivation drift is still significantly larger than 0 for most of the time points. Meanwhile, the same value in neural data (Fig. 5d) seems insignificant from 0. I'm wondering how the authors interpret this discrepancy.
3. In the introduction (line 45), the authors mentioned "While V1 neural receptive fields are strictly contralateral". This might be too absolute; replacing the "strictly" with "relatively" could be better.
4. In the model simulation, the prefrontal hemispheres are connected by tuned interhemispheric connections. Since the authors have simultaneous bilateral prefrontal neural data, have they checked these tuned interhemispheric connections in the real data?
5. Several possible incorrect statements:
In line 144, "Sa" may be mistaken for "SD";
In line 271, "the within-area connectivity" might be "across-area";
In line 928, it is stated that "the acquisition rate of the eye tracker and electrophysiology was the same." Usually, the acquisition rate of electrophysiology (40kHz or less) is much larger than that of the eye tracker (1kHz). Here, the authors might refer to the processed acquisition rate of neural data, rather than the raw acquisition rate of the recording signal.

(Remarks on code availability)

Reviewer #3

(Remarks to the Author)
This study investigates how interhemispheric organization supports spatial working memory by combining bilateral prefrontal recordings in macaques and attractor network modeling. In the experiment, the authors find that both hemispheres represent the full visual space with a contralateral bias, equally predict behavioral errors, but show weakly correlated memory coding and non-correlated (private) reactivation. They interpret this as a redundant architecture with weak interhemispheric coupling, and use computational modeling to argue this organization adaptively balances memory precision and capacity depending on task demands. The bilateral prefrontal cortex recording dataset is impressive, the manuscript is clearly written, and the analyses are, for the most part, rigorous and well motivated. With that said, the main conceptual advance that this weakly connected architecture “reconciles” hemispheric specialization vs. redundancy theories relies heavily on computational predictions rather than empirical evidence. However, the insights are appealing.
Below are my major comments. These concerns do not diminish the value of the bilateral recording data or the basic findings about interhemispheric coupling, but some additional analyses and clarification would strengthen the interpretation.
1. As said above, the current data convincingly demonstrate redundant and weakly coupled hemispheric representations but do not directly test task-dependent switching between redundant and specialized modes. The main claims about how the prefrontal hemispheres “adapt storage strategies to task demands” or how this work “reconciles” competing frameworks are derived entirely from simulations. I recommend softening these statements in abstract, results, and even title, and clearly distinguishing what is shown empirically vs. what is inferred from the model. Wording such as “the model suggests” would better reflect the evidence. The multi-item simulation (Fig. 6) makes interesting predictions about the bilateral field advantage, but this should be explicitly framed as a prediction requiring future experimental testing rather than as evidence supporting the theory.
2. The finding of redundant hemispheric representations could reflect uniform full-field redundancy or redundancy primarily near the vertical meridian, where interhemispheric projections are denser. The latter would be more consistent with known anatomy and would change how we interpret “redundancy” - from a fail-safe backup system to a mechanism for ensuring smooth representation across the hemispheric boundary. A supplementary spatial analysis comparing midline vs. peripheral stimuli would clarify this point.
The model also assumes uniform coupling across space (though tuned by receptive field similarities) and implements a sharp hemispheric boundary at the vertical meridian, which seems biologically implausible. A model with topographically graded connectivity would be more realistic and might make different predictions.
3. The across-hemisphere error correlations are central to the weak coupling conclusion. However, these correlations could potentially arise from shared noise sources rather than interhemispheric communication. While the authors rule out eye movements as a confound, other global sources of variance (e.g., arousal, trial difficulty, or slow firing-rate drifts) could still jointly affect decoding and behavioral errors. It would be helpful either to include additional control analyses (pupil size, previous reward, etc.) or to discuss how such covariates might influence the results.
4. The manuscript does not specify whether error or outlier trials were excluded and, if so, what criteria were used. This information is important because large-error trials could strongly influence behavioral variance, serial dependence estimates, and decoder-behavior correlations. If trials with large errors were excluded (e.g., saccades landing far from target), this could remove exactly the variance needed for several key analyses. Please clarify whether such trials were included and, if possible, confirm that the main results remain robust when they are included/excluded or when different criteria are used.
Likewise, it is not specified if all units are used in decoding analyses, or only selective units. It is important to briefly clarify these details in the method or even in the main text.
5. One subject (Pe) shows strikingly different patterns from the other animals: little or no serial dependence and much weaker memory coding. Pe is consequently excluded from the key reactivation analyses, leaving n=2 for these findings. Since this variability is substantial, it would be useful to discuss whether it reflects data quality differences, lower task engagement, individual strategic differences, or simply noisier memory encoding. Clarifying this point would help readers interpret the generality of the observed effects across subjects.
6. Given the emphasis on bilateral equivalence, it would be helpful to examine whether results hold when trials are analyzed separately for left- and right-sided stimuli. A supplementary figure showing behavioral precision, serial dependence, and decoding–behavior correlations by hemifield would help rule out side-related asymmetries that might influence the redundancy interpretation.
7. The equal behavioral prediction from ipsilateral and contralateral decoding is interpreted as evidence for redundancy. However, an alternative explanation is that this reflects downstream motor integration rather than redundant memory coding. If each hemisphere has a biased but partial representation, the motor system might integrate these biased signals to produce behavior, resulting in equal predictive power even without full redundancy in the memory representations themselves.
Below are some minor points:
1. “BIC” should be defined on first use.
2. “Area 8Ar”: briefly note this is dorsolateral prefrontal for readers unfamiliar with macaque nomenclature cortex and note its role in spatial working memory.
3. The 30% threshold of reactivation strength appears arbitrary. Please clarify the rationale for choosing the threshold.
4. Extended Data Fig. 7 misses label for panel C on the figure.
5. Missing statistics (p value) for Fig 6 simulation results.
6. Mixed use of PFC and prefrontal cortex. Please pick one for consistency.

(Remarks on code availability)
Code is prepared in clear structure and well documented. Cannot test the code since data is not available yet.

Version 1:

Reviewer comments:

Reviewer #1

(Remarks to the Author)
I thank the authors for their thorough and careful response to my comments. They have addressed all of my concerns. I am impressed with the quality of the work and the rigor of the revised submission.

(Remarks on code availability)

Reviewer #2

(Remarks to the Author)
The authors have answered all my questions.

(Remarks on code availability)

Reviewer #3

(Remarks to the Author)
Thank you for the careful and thorough revisions. The added results substantially strengthen the manuscript and transform the paper from presenting a speculative model to providing empirical validation of key claims. In figure 6g, a clever analysis on the existing monkey data demonstrates better behavioral precision with dual-hemisphere accuracy. In figure 7, analyses on human multi-item memory data directly supports the load-dependent switching between redundant and specialized storage. There are only a couple remaining minor points that may require additional clarification.

[Re: Point 3] The added discussion text addresses this well. One minor point: correlated noise from shared upstream visual areas (V4, LIP) could create spatially structured perturbations similar to direct callosal connections. This doesn't undermine your conclusions as both indicate interhemispheric coordination, but you might briefly acknowledge this alternative circuit mechanism.

[Re: Point 4] Thanks for adding in the criteria for trial removal. Does the term “consecutive correct trials” mean that the n-th trial and the (n-1)-th trial should both be correct to be included in the analyses? Since this might influence the serial dependence results (e.g., under-estimating the effect due to reduced variance, or cleaning up the pattern with outliers removed), it would be helpful to include a short explanation for the choice to include only consecutive correct trials.

[Re: Point 7] Figure 6g may actually provide additional evidence for your interpretation: if equal behavioral prediction reflected only motor integration of biased estimates, you wouldn't expect better precision when both hemispheres are accurate versus only one. This reinforces that redundancy is in memory representations themselves, not just motor readout. Worth briefly noting when discussing Fig. 6g.

I recommend accepting the paper with (optional) slight revision as discussed above.

(Remarks on code availability)

Open Access This Peer Review File is licensed under a Creative Commons Attribution 4.0 International License, which permits use, sharing, adaptation, distribution and reproduction in any medium or format, as long as you give appropriate credit to the original author(s) and the source, provide a link to the Creative Commons license, and indicate if changes were made.
In cases where reviewers are anonymous, credit should be given to 'Anonymous Referee' and the source.
The images or other third party material in this Peer Review File are included in the article's Creative Commons license, unless indicated otherwise in a credit line to the material. If material is not included in the article's Creative Commons license and your intended use is not permitted by statutory regulation or exceeds the permitted use, you will need to obtain permission directly from the copyright holder.
To view a copy of this license, visit https://creativecommons.org/licenses/by/4.0/

Rebuttal letter: Redundant prefrontal hemispheres adapt storage strategy to working memory demands

We thank the three reviewers for their careful reading of our manuscript and their insightful comments. We modified our manuscript accordingly and made significant additions to address all their comments. This has resulted in a significant improvement of our manuscript.

In the following, we detail point-by-point all the changes introduced in the manuscript and our specific responses to the reviewers' questions and comments:

Reviewer #1:

In this manuscript, the authors combine neural recordings in monkeys performing a visuo-spatial working memory task with artificial neural network (ANN) modeling to investigate how working memory (WM) is distributed across hemispheres. Using bilateral recordings in lateral prefrontal cortex (lPFC, area 8Ar) and population decoding, they replicate previous findings that both hemispheres represent the location of the target during the delay, with stronger representation in the contralateral hemi-field. Additionally, they demonstrate that in both hemispheres, population decoder errors (clockwise (CW) and counterclockwise (CCW)) correlate with behavioral errors, indicating that both hemispheres could jointly support behavior. However, this error correlation was stronger within than across hemispheres, suggesting that WM representations are weakly coupled between hemispheres. This finding is corroborated by their ANN modeling, which additionally predicted that serial dependency is hemisphere-specific in their neural recordings. Finally, they show that in their two weakly coupled single-item capacity ring attractor network model, adding a second item leads to lateralization. From this alone, they conclude that storage strategy (lateralized vs. redundant) depends on cognitive demand.

Overall, this manuscript combines neural data analyses with ANN modeling to address an interesting question in the field—how spatial WM is shared across hemispheres. The ANN model makes specific predictions about error correlations and the effect of trial history within and across hemispheres that are corroborated by their neural data analyses. These results are convincing and clearly presented. My main concern is that their result on cognitive demand appears specific to their modeling choice and cannot be tested with their data.

Although this is their weakest result, they rely heavily on it in their conclusion (and even the title), which diminishes my enthusiasm for the paper. Below, I expand on this concern and other aspects of the manuscript.

We would like to thank the reviewer for their helpful and detailed feedback and for sharing their concerns. We now provide additional empirical data supporting the claims of our manuscript in relation to cognitive demands. Specifically, we now show that dual maintenance in monkey hemispheres (i.e. redundant storage) leads to more precise behavior (Fig. 6g) and that a switch from redundant to specialized storage can be observed when going from low to high memory loads in an openly available human dataset of multi-item working memory, as predicted by a higher-capacity version of the model that we originally proposed (Fig. 7).

As stated above, my primary concern regards the final section (Figure 6), where the authors show that in their two weakly coupled single-item capacity ring attractor network model, adding a second item and a lateralization signal leads to: (i) contralateral memory specialization and (ii) reduced memory capacity for items presented in the same hemi-field.

I don’t believe that a single-item capacity ANN is an appropriate model of WM for multiple stimuli. Since the ANN cannot hold two items within the same hemisphere, it seems trivial that when presented with two items, it will forget one and is more likely to forget the stimulus with the weaker input (from the ipsilateral hemifield). I believe a more realistic model with multiple-item capacity and account for interference would be more appropriate and will make more realistic predictions (e.g., references 37-41 and Compte et al., 2000; and see below for why a pure slot model is unlikely to be a good model for lPFC).

This is a valid point. We have included an additional Figure (Fig. 7) implementing a network model with higher capacity. We modified some parameters of the model from Fig. 6 to allow for the simultaneous storage of multiple items in each area (Fig. 7a-e). In this extended model, items can be maintained, merged, or die out (Fig. 7a), leading to the requested, more realistic multi-item working memory dynamics (Edin *et al.*, 2009; Wei, Wang and Wang, 2012; Almeida, Barbosa and Compte, 2015).

As the reviewer pointed out, these multi-item networks are susceptible to memory interference. In particular, representations may drift towards nearby non-targets when multiple stimuli are present. This property allows the model to generate further predictions regarding the interaction of memory interference and stimulus laterality under varying task demands.

To generate these predictions, we simulated low-load (presenting 2 items) and high-load (presenting 4 items) conditions. Under low load, both areas mostly stored items redundantly.

Under high loads, each area specialized for its contralateral items, leading to a bilateral field advantage (Fig. 7b).

Importantly, the higher-capacity model predicts that attraction towards non-targets is independent of the item hemifields for low loads, due to redundant storage (Fig. 7 c,e, Supplementary Fig. 10b). On the contrary, under high load, non-target attraction is reduced when the non-target is presented in the opposite hemifield, reflecting the contralateral specialization (Fig. 7 d,e, Supplementary Fig. 10c). We confirmed this model prediction in a behavioral human data set (Figure 7 f-j).

Thus, the higher-capacity model recapitulates and extends the principle illustrated in the single-item network (Fig. 6), in a more realistic setting. We retain Fig. 6 as an intuitive presentation of the general principle we found in the monkey data, but also explicitly simulate and test human multi-item working memory in Fig. 7.

The main weakness of these results is that the specific prediction that their model makes (e.g., one item is randomly forgotten when two items are presented in the same hemifield if the other one is redundantly represented) cannot be tested with their data, as they do not manipulate the load. Additionally, a pure slot model is unlikely to be a good model for WM in lPFC, as information about items in working memory has been shown to be reduced when more items are added in the same hemi-field rather than being 'all or none' (Buschman et al., 2011).
Overall, this is their weakest result, and I do not believe it should feature so prominently in their interpretation. In particular, the title is misleading.
We addressed the reviewer’s very reasonable concerns in different ways. For one, we demonstrated that our computational model is not “a pure-slot model” and is consistent with existing physiology data (1 below). Furthermore, we found a way to experimentally test the predictions of our model in a multi-item WM task (2 below). We also validated our computational model's prediction that redundant storage improves precision (3 below).

1) Our computational network model (Fig. 7a) is actually consistent with existing neurophysiological reports in multi-item working memory and does not show an “all-or-none” mode of operation. We plotted firing rates for target-selective neurons when a non-target item was presented in the same or in the other area, and verified reduced firing when the non-target was in the same area (Supplementary Fig. 10a). This is consistent with previous data showing that adding an item in the same but not the opposite hemifield reduces decodability of the initial target stimulus (Buschman *et al.*, 2011; Matsushima and Tanaka, 2014). We want to emphasize that attractor

network models proposed to explain multi-item working memory do not typically adhere to the “slots model” idea. Mean network activity does not linearly scale with load, but markedly sublinearly (Edin *et al.*, 2009; Wei, Wang and Wang, 2012; Almeida, Barbosa and Compte, 2015)).

2) We can now empirically show a change from redundant to specialized hemifield storage as memory load increases in working memory tasks. We reanalyzed behavioral data from two publicly available human experiments (Schneegans and Bays, 2016, 2018) (Fig. 7f-j), in which participants were asked to memorize multiple spatial locations over a short delay. We analyzed the errors of the reported location in relation to the memory load, the distance to other memoranda, and hemifield locations, following the analysis derived from our network simulations (Fig 7c-e, see reply to previous comment). The proposed switch in storage strategy between redundancy at low load and specialization at high load was indeed reflected in errors committed in the multi-item task. Specifically, attraction to other memoranda within and across hemifields only differed at high but not at low loads, even when controlling for target-nontarget distance. This is consistent with redundant storage at low loads and specialization at high loads, as predicted by the model, and supports our interpretation of the results.
3) We can further demonstrate empirically, using the monkey dataset, that redundant storage improves precision in spatial working memory. We split trials of the single-item monkey experiments based on whether the neural populations in both hemispheres show an accurate representation of the item at the end of the delay, or whether either hemisphere randomly forgot the item. We found that the behavioral precision of the monkey was indeed improved when items were stored redundantly in both hemispheres (Fig. 6g). This analysis confirms the prediction of the model that redundant storage improves precision.

We believe that these additional analyses, backed up with empirical validation of model predictions, strengthen the manuscript’s message significantly. We want to thank the reviewer for raising a reasonable concern and prompting us to find stronger evidence supporting our claims.

I find it difficult to reconcile the findings described in Figure 2h (greater decoding accuracy of the target location for the contralateral hemi-field) and Figure 3c (no difference in the brain-behavior correlation on a trial-by-trial basis). The authors conclude that both hemispheres are equally involved in guiding behavior. It appears that the analysis in Figure 3c is noisier, and the absence of difference between the hemi-fields represents a null effect.

We understand the reviewer's concern regarding this analysis. Instead of highlighting that there is no significant difference between areas, we changed the text to highlight that both hemispheres show significant correlation with behavior. The model that we are trying to confront is one in which, despite redundant storage, behavior is only determined by contralateral representations. Significant correlation in both hemispheres already discards this model, so across-hemisphere comparison is not required.

In order to strengthen this point, we also performed an additional analysis: We only used trials in which ipsilateral and contralateral error predictions had opposite signs (Supplementary Fig. 5c). This analysis showed no clear sign of either hemisphere dominating when representations disagreed, suggesting that neither hemisphere dominates the behavioral process.

Changes:

“Importantly, activity from either hemisphere could guide bilateral behavior, consistent with redundant memories.” (lines 102-103)

“Similarly, both ipsilateral and contralateral trials significantly correlated with behavior (Fig. 3c, Supplementary Fig. 5a). Interestingly, when we exclusively considered trials in which hemispheric predictions were in opposite directions (clockwise vs. counterclockwise), neither hemisphere dominated the correlation with behavior (Supplementary Fig. 5c). Therefore, even though the memory code was more precise in contralateral trials (Fig. 2g), both hemispheres seemed of similar importance for the behavior.” (lines 183-188)

“This analysis showed that both hemispheres similarly predicted small behavioral imprecisions in the full visual field and were both weighted in guiding behavior. Therefore, in combination with the strong ipsilateral decoding (Fig. 2h), our analyses did not support the specialized model (Fig. 1a), but rather pointed toward a redundant architecture (Fig. 1b), with both hemispheres encoding stimuli and guiding behavior in the full visual field.” (lines 189-193)

Figure 3e: Error correlation is significantly above zero before stimulus onset (also in Figure S5c). Can the authors explain this? This is particularly concerning for the 'within' curve, which does not seem be above this 'baseline.'

We thank the reviewer for pointing this out. When reviewing the code, we found that we were using a uniform correction for the circular correlations (Vallat, 2018), a remnant from correlating prediction angles (which were uniformly distributed on the circle across trials, so the correction was indeed necessary) in a previous analysis (not used in the current manuscript). Since the errors are not uniformly distributed, we were mistaken in using this correction and now refrain from using it. This correction only had a relevant impact in periods

with very weak decoding, in particular in the fixation period. We corrected the code, the Methods section, the statistics, and the Supplementary Material in the text. Based on this corrected method, the correlation before stimulus onset is no longer significantly different from zero in the across-hemisphere condition (Fig. 3e). For the within-hemisphere condition, Fig. 3e still shows significantly positive error correlation in the fixation period, which is consistent with private reactivations in fixation, as we explore in detail in Fig. 5.

Minor Comments

• Figure 1d is difficult to read. I recommend using a color gradient and making this panel larger (as in Wimmer et al., 2014).
Done.
• Model lines are missing from Figure 1f.
Done.

• Monkey Pe has a very low number of multi-units that are selective for a location during the delay compared to the two other monkeys (Figure 2a legend). Can the authors explain this?
As far as we can tell, this reflects only a sampling difference of the implanted array. We now specify this further:
"59.13% (3375) of 5708 multi-units were selective during the delay (Sa: 88.4% (1950 of 2206 selective units), Pe: 28.1% (666 of 2370 selective units), Wa: 72.23% (705 of 976 selective units), bootstrapped test, see Methods, differences likely due to sampling differences of the implanted arrays)." (Caption of Fig. 2c, lines 1219-1221)

"Monkey Pe tended to have a smaller amount of selective neurons in each session (average of 45 selective units per session) than the other monkeys (121 (monkey Sa) and 78 (monkey Wa) selective units per session), likely due to sampling differences of the implanted array." (lines 535-537)

• Figure 2h and lines 190/198, page 8: The authors state that memory reactivation has been previously shown to correlate with serial dependence. Can they demonstrate this in their data as well?
No, we could not replicate the effect in this data set. When we split trials based on the absolute reactivation error into trials with strong and weak reactivations (median split for each session), we cannot find a significant difference between their respective behavioral serial dependence curves (see figure).

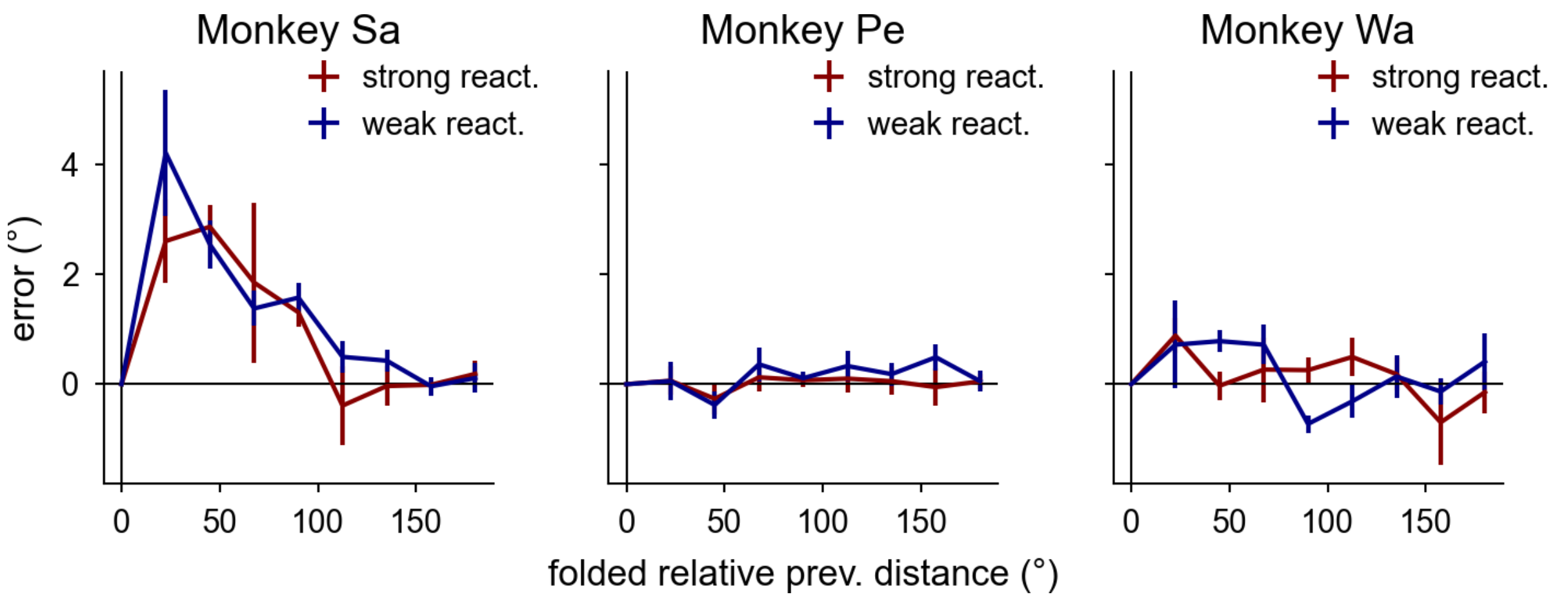

Behavioral serial dependence split for precise (strong) and imprecise (weak) previous reactivations for the three monkeys.

Statistically, we tested this effect with a linear mixed model, fitting the current error (err_{curr}) with the DoG1-predicted serial bias ($DoG1(\Delta_{prev,curr})$), the absolute reactivation error ($abs(err_{react})$), and their interaction (which would be the regressor of interest), while accounting for monkey-based differences in serial dependence through random effects on the intercept and the serial bias:

$$err_{curr} \sim \beta_0 + \beta_{DoG} DoG1(\Delta_{prev,curr}) + \beta_{react} abs(err_{react}) + \beta_{interact}(DoG1(\Delta_{prev,curr}) \cdot abs(err_{react}))$$

We found no significant interaction ($\beta_{interact}$=0.1°, p=0.12).

We have added a line to the main text:
"We could not replicate this effect here, which may be due to varying, shorter inter-trial intervals, variable delays or other differences in the experimental design." (lines 165-167)

• Lines 202-207, page 8: It took me some time to realize that the behavioral and neural decoder errors were signed (CW/CCW). Could the authors make this clearer in the text?
Done. We now write: "To address how PFC activity related to behavioral responses, we correlated the signed (clockwise, counterclockwise) neural decoder errors with the corresponding signed behavioral response errors" (lines 174-175)
• Lines 202-207, page 8: Why use the slope as your statistical test for correlation? It seems that a significant correlation is what matters (as an area under the curve if the timing is uncertain).

We used the slope because the prediction of a memory diffusion model is explicitly a continuously growing correlation over the delay period (Wimmer *et al.*, 2014). It is thus a stronger prediction than the mere correlation, which could, for instance, reflect the efference copy of a motor command issued by another brain area. Thus, while taking the area under the curve of the entire memory period would also validate some assumptions of the model, it would not fully capture the dynamics of the effect predicted. However, for completeness, we also performed this analysis. We now took AUC at the end of the delay, using the last 3 time bins (reflecting 600ms), and found significance for all trials, ipsilateral and contralateral trials (All: $t(39) = 6.36$, $p = 1.63e-07$, mean = 0.11, 95% CI [0.07, 0.14] fit on 3 independent 0.2s time bins. Ipsi: AUC 1-sample: $t(8) = 3.43$, $p = 8.95e-03$, mean = 0.13, 95% CI [0.04, 0.22] fit on 3 independent 0.2s time bins, Contra: AUC 1-sample: $t(8) = 3.44$, $p = 8.76e-03$, mean = 0.15, 95% CI [0.05, 0.25] fit on 3 independent 0.2s time bins). However, we still feel that using the slope reflects the steady increase in correlations that the computational model explicitly predicts.

Reviewer #2:

The authors combine electrophysiological recordings from macaque monkeys performing a visuospatial task and neural network simulation to elucidate two alternative mechanisms of interhemispheric coding for working memory across the entire visual space: specialized coding vs. redundant coding. In electrophysiological recordings, the authors performed simultaneous recordings on the bilateral prefrontal cortex of a monkey and found redundant coding in each hemisphere with weak coupling; in modeling, they not only replicate the main findings from the experiments but also demonstrate the switch between specialized coding and redundant coding, which depends on the task demand.

This is a very interesting and systematic work. The authors carefully select the within-trial imprecision and the across-trial serial dependence, both of which are closely related to working memory coding and operate on different time scales. Based on the experimental findings, they construct a redundant, weakly connected network with lateralized inputs, which ingeniously reconciles the two interhemispheric coding mechanisms.

We would like to thank the reviewer for their positive feedback and for the concerns raised below. We agree with the mentioned points and have updated the manuscript accordingly.

I just have some points which may be helpful to improve the manuscript:

1. I noticed that in neural data, the across-area error correlation seems significantly larger than 0 before the stimulus presentation (Fig. 3e), while the same quantity is close to 0 in the

model (Fig. 4e). I'm wondering how the authors interpret this discrepancy. I think explaining the non-zero across-area error correlation in neural data is important, as this is the major evidence supporting interhemispheric correlations.

We thank the reviewer for pointing this out. When reviewing the code, we found that we were using a uniform correction for the circular correlations (Vallat, 2018), a remnant from correlating prediction angles (which were uniformly distributed on the circle across trials, so the correction was indeed necessary) in a previous analysis (not used in the current manuscript). Since the errors are not uniformly distributed, we were mistaken in using this correction and now refrain from using it. This correction only had a relevant impact in periods with very weak decoding, in particular in the fixation period. We corrected the code, the Methods section, the statistics, and the Supplementary Material in the text. Based on this corrected method, the correlation before stimulus onset is no longer significantly different from zero in the across-hemisphere condition (Fig. 3e). For the within-hemisphere condition, Fig. 3e still shows significantly positive error correlation in the fixation period, which is consistent with private reactivations in fixation, as we explore in detail in Fig. 5.

2. In the model with weak across-area connectivity (Fig. 3h), the authors found that the across-hemisphere reactivation drift is much smaller than the within-hemisphere one. However, it seems that this across-hemisphere reactivation drift is still significantly larger than 0 for most of the time points. Meanwhile, the same value in neural data (Fig. 5d) seems insignificant from 0. I'm wondering how the authors interpret this discrepancy.

We can think of two potential interpretations of this discrepancy:

1) There might be a small positive across-hemisphere reactivation drift, but the data is unable to detect it significantly based on experimental limitations: number of recorded neurons, experimental noise, etc.
2) Dual reactivations may be more frequent in our network simulations (~15% of trials, Fig 4k) than in the experiment. A lack of dual reactivations in the data would explain why no across-hemisphere attraction emerges.

We added this to the manuscript:

"Across-hemisphere drift was somewhat weaker than in the simulations, probably reflecting either lower incidence of dual reactivations or large noise levels, hiding a weak effect." (lines 308-310)

3. In the introduction (line 45), the authors mentioned "While V1 neural receptive fields are strictly contralateral". This might be too absolute; replacing the "strictly" with "relatively" could be better.

Done, replaced with "almost exclusively".

4. In the model simulation, the prefrontal hemispheres are connected by tuned interhemispheric connections. Since the authors have simultaneous bilateral prefrontal neural data, have they checked these tuned interhemispheric connections in the real data?

This analysis has indeed been performed in a manuscript that uses the same data (McDonnell *et al.*, 2025). Their Supplementary Fig. S2 shows that during the delay period, noise correlations (r_{sc}) of across-hemisphere neuron pairs are positive when neurons have similar tuning, and negative when neurons have opposite tuning. This suggests tuned interhemispheric connections in the data. We highlighted this in the text:

"Our observation of correlated neural errors between the hemispheres led us to infer the existence of weak, tuned interhemispheric connections, consistent with observed interhemispheric noise correlations in these data (see Fig. S2 in (52))" (lines 471-473)

5. Several possible incorrect statements:

In line 144, "Sa" may be mistaken for "SD";

Sa refers to the name of the monkey, not serial dependence. We changed this in the text to reflect the distinction more clearly.

In line 271, "the within-area connectivity" might be "across-area";

We indeed mean that the inter-area connectivity is expressed as a percentage of the "within-area connectivity". Since the wording was misleading, we changed it to:

"We studied the effects of different interhemispheric interactions by manipulating the strength and structure of excitatory connections between the two areas (Fig. 4a), expressed as a percentage of the excitatory coupling strength within each area (see Methods)." (line 232-234).

In line 928, it is stated that "the acquisition rate of the eye tracker and electrophysiology was the same." Usually, the acquisition rate of electrophysiology (40kHz or less) is much larger than that of the eye tracker (1kHz). Here, the authors might refer to the processed acquisition rate of neural data, rather than the raw acquisition rate of the recording signal.

Yes, the reviewer is correct. We corrected this information in the manuscript:

"Since the acquisition rate of the eye tracker and the used, preprocessed acquisition rate of the electrophysiology (original acquisition rate 30kHz, downsampled to 1 kHz) were the same, we binned the eye locations in the same manner as the neural data." (lines 687-689)

Reviewer #3:

This study investigates how interhemispheric organization supports spatial working memory by combining bilateral prefrontal recordings in macaques and attractor network modeling. In the experiment, the authors find that both hemispheres represent the full visual space with a

contralateral bias, equally predict behavioral errors, but show weakly correlated memory coding and non-correlated (private) reactivation. They interpret this as a redundant architecture with weak interhemispheric coupling, and use computational modeling to argue this organization adaptively balances memory precision and capacity depending on task demands. The bilateral prefrontal cortex recording dataset is impressive, the manuscript is clearly written, and the analyses are, for the most part, rigorous and well motivated. With that said, the main conceptual advance that this weakly connected architecture "reconciles" hemispheric specialization vs. redundancy theories relies heavily on computational predictions rather than empirical evidence. However, the insights are appealing.

Below are my major comments. These concerns do not diminish the value of the bilateral recording data or the basic findings about interhemispheric coupling, but some additional analyses and clarification would strengthen the interpretation.

We would like to thank the reviewer for their helpful insights on the manuscript. The call for clarification and improvement is well motivated, and we addressed the posed issues, as we detail below. In particular, we have included new data and simulations to address these concerns.

1. As said above, the current data convincingly demonstrate redundant and weakly coupled hemispheric representations but do not directly test task-dependent switching between redundant and specialized modes. The main claims about how the prefrontal hemispheres "adapt storage strategies to task demands" or how this work "reconciles" competing frameworks are derived entirely from simulations. I recommend softening these statements in abstract, results, and even title, and clearly distinguishing what is shown empirically vs. what is inferred from the model. Wording such as "the model suggests" would better reflect the evidence. The multi-item simulation (Fig. 6) makes interesting predictions about the bilateral field advantage, but this should be explicitly framed as a prediction requiring future experimental testing rather than as evidence supporting the theory.

We thank the reviewer for pointing out this crucial missing step in our manuscript. This concern was also raised by Reviewer #1, and we have addressed it by presenting additional empirical data that validate the predictions of the model, now presented in Fig. 6g and in a new Fig. 7.

We now provide two new pieces of evidence that support the model interpretation: (1) redundant storage results in more precise working memory, which we show by analyzing monkey behavior for trials in which neural representations of the memorized item are accurate in both hemispheres (redundant storage), and compare it with behavior for trials in which at least one hemisphere does not have an accurate neural representation. As

predicted by the model, monkey responses were more precise for the redundant storage condition (Fig. 6g); (2) in human multi-item spatial working memory (Fig. 7f), distance-conditioned attraction of the target memory to a non-target item is independent of whether non-targets appear in the same or opposite hemifield as the target for low-load trials (redundant storage). However, for high loads, across-hemifield attraction is much weaker than within-hemifield attraction, suggesting specialized storage (Fig. 7h-j). This supports a change in hemispheric storage strategy based on cognitive demands (high vs low load) experimentally, as predicted by a high-capacity extension of the model (Fig. 7a-e).

2. The finding of redundant hemispheric representations could reflect uniform full-field redundancy or redundancy primarily near the vertical meridian, where interhemispheric projections are denser. The latter would be more consistent with known anatomy and would change how we interpret "redundancy" - from a fail-safe backup system to a mechanism for ensuring smooth representation across the hemispheric boundary. A supplementary spatial analysis comparing midline vs. peripheral stimuli would clarify this point.
Following the reviewer's suggestion, we performed a new analysis by separately correlating interhemispheric decoder errors for trials with stimuli presented on the vertical meridian (90° or 270°, *midline*, orange) or along the horizontal line (0° or 180°, *periphery*, red). We found no evidence for stronger redundancy along the vertical meridian (Supplementary Fig. 6e) and thus no support for a redundant model specific to the vertical meridian.

The model also assumes uniform coupling across space (though tuned by receptive field similarities) and implements a sharp hemispheric boundary at the vertical meridian, which seems biologically implausible. A model with topographically graded connectivity would be more realistic and might make different predictions.
We apologize for the confusion. The network model does not have a sharp hemispheric boundary regarding its coupling. The only interhemispheric boundary implemented is that of the lateralized inputs. However, this laterality is supported by the strong laterality in V1, which we therefore kept for simplicity.
It is true, though, that we could implement different coupling for targets placed on the midline, for example. However, the additional analysis described in the previous point did not indicate a change, so we kept this architecture uniformly distributed. We added a paragraph in the Methods:
"Tuning was defined solely based on the distance between preferred locations of pre- and post-synaptic neurons across the two area networks. An alternative tuning function may additionally depend on the preferred location of the presynaptic neuron, which would allow to model midline connectivity inhomogeneities. However, our analysis of midline versus

peripheral error correlations did not show any evidence for midline specificity (Supplementary Fig. 6e), and we therefore did not investigate this connectivity structure further." (lines 783-788)

3. The across-hemisphere error correlations are central to the weak coupling conclusion. However, these correlations could potentially arise from shared noise sources rather than interhemispheric communication. While the authors rule out eye movements as a confound, other global sources of variance (e.g., arousal, trial difficulty, or slow firing-rate drifts) could still jointly affect decoding and behavioral errors. It would be helpful either to include additional control analyses (pupil size, previous reward, etc.) or to discuss how such covariates might influence the results.

We agree with the reviewer that we did not include all potential sources of shared variability. However, we want to emphasize that the error correlations that we are considering are of a very specific nature that are not conceivably affected by other behavioral or task covariates, such as arousal, trial difficulty, or slow firing-rate drifts. In particular, for decoder errors in the two hemispheres to be jointly driven by a common input, this shared input would have to be aligned with the retinotopic space of stimulus memory and induce consistent small clockwise vs counterclockwise decoder errors. The most obvious source for such tightly spatially tuned correlates is that of eye movements, which we have accounted for. Based on this, we think it is reasonable to assume that this supports some weak communication between the two hemispheres.

Additionally, this conclusion was also reached in another study using the same data (McDonnell *et al.*, 2025), focusing on non-task-related covariation in neural activity. Their analysis showed stronger noise correlations for similarly tuned neurons across hemispheres, also suggesting tuned interhemispheric connections.

Finally, when viewing the results of Fig. 4, we would like to highlight that even without tuned interhemispheric connections, the results for private serial dependence would remain intact, since the 0.0% connected and 0.2% connected models do not differ qualitatively. We added a paragraph in the discussion to clarify this concern:

"Our observation of correlated neural errors between the hemispheres led us to infer the existence of weak, tuned interhemispheric connections, consistent with observed interhemispheric noise correlations in these data (see Fig. S2 in (54)). While one may argue that these correlations rather reflect common input, such input would need to induce consistent rotations of mnemonic representations in both hemispheres to explain the alignment of small memory imprecisions. Uninstructed eye movements, the most plausible confound, did not alter the effect. Moreover, even if an unidentified confound contributed, our model predictions beyond shared memory diffusion should remain largely intact, since the

unconnected and weakly connected models from Fig. 4 show no qualitative differences." (lines 471-479)

4. The manuscript does not specify whether error or outlier trials were excluded and, if so, what criteria were used. This information is important because large-error trials could strongly influence behavioral variance, serial dependence estimates, and decoder-behavior correlations. If trials with large errors were excluded (e.g., saccades landing far from target), this could remove exactly the variance needed for several key analyses. Please clarify whether such trials were included and, if possible, confirm that the main results remain robust when they are included/excluded or when different criteria are used.
Thank you for highlighting this. We clarified this in the Methods:
"We excluded all trials with fixation breaks and only utilized rewarded trials (29,459 trials out of 64,504 trials were rewarded (45.67% rewarded trials)). Furthermore, we excluded trials with errors larger than 22.5° (291 trials excluded, ~1%) and only analyzed consecutive correct trials, leaving a total of 18,184 trials (61.72% of all rewarded trials)." (lines 515-518)
Likewise, it is not specified if all units are used in decoding analyses, or only selective units. It is important to briefly clarify these details in the method or even in the main text.
Thank you for highlighting this. We have added it in the Methods (lines 1001-1002):
"We excluded multi-units with firing rates lower than 1 Hz for the analyses."(lines 533-534)

5. One subject (Pe) shows strikingly different patterns from the other animals: little or no serial dependence and much weaker memory coding. Pe is consequently excluded from the key reactivation analyses, leaving n=2 for these findings. Since this variability is substantial, it would be useful to discuss whether it reflects data quality differences, lower task engagement, individual strategic differences, or simply noisier memory encoding. Clarifying this point would help readers interpret the generality of the observed effects across subjects.
Overall, serial dependence seems to be less stereotypical in monkeys than in humans, and even humans show idiosyncratic differences in serial dependence strength (Stein et al., 2020; Zhang and Alais, 2020; Kim and Alais, 2021). While monkey Pe showed no serial dependence, this monkey had the highest memory precision (see Fig. 1e), suggesting good behavioral performance and task engagement. On the other hand, the monkey had the least selective neurons recorded from the recording array, but as far as we can tell, this only reflects a difference in the neural sampling obtained from the implanted arrays in different monkeys.
While monkey Pe was excluded from the neural serial dependence analyses for these reasons, we did find that overall serial dependence in behavior tended to correlate with

neural serial dependence, though more subjects are needed to establish this effect at a population level (Supplementary Fig. 8a).

We added this information to the manuscript:

"We found varying degrees of serial dependence across monkeys (Fig. 1f), akin to known idiosyncratic differences in humans (50, 68, 69)" (lines 133-135)

"59.13% (3375) of 5708 multi-units were selective during the delay (Sa: 88.4% (1950 of 2206 selective units), Pe: 28.1% (666 of 2370 selective units), Wa: 72.23% (705 of 976 selective units), bootstrapped test, see Methods, differences likely due to sampling differences of the implanted arrays)." (Caption Fig. 2a, lines 1219-1221)

lines 1003-1004:

"Monkey Pe tended to have a smaller amount of selective neurons in each session (average of 45 selective units per session) than the other monkeys (121 (monkey Sa) and 78 (monkey Wa) selective units per session), likely due to sampling differences of the implanted array." (lines 535-537)

6. Given the emphasis on bilateral equivalence, it would be helpful to examine whether results hold when trials are analyzed separately for left- and right-sided stimuli. A supplementary figure showing behavioral precision, serial dependence, and decoding–behavior correlations by hemifield would help rule out side-related asymmetries that might influence the redundancy interpretation.

We added a supplementary figure for the effect of left vs right hemifield stimuli on precision (Supplementary Fig. 1a) and serial dependence for the previous stimulus (Supplementary Fig. 1b-d, top) and the current stimulus (Supplementary Fig. 1b-d, bottom). This adds to the left vs. right separation for the decoding-behavior correlations (Fig. 3b, Supplementary Fig. 5b) and completes the full view of laterality effects in this data set. In short, we do not find evidence for any lateral specialization (left-right) in spatial working memory in our monkeys.

7. The equal behavioral prediction from ipsilateral and contralateral decoding is interpreted as evidence for redundancy. However, an alternative explanation is that this reflects downstream motor integration rather than redundant memory coding. If each hemisphere has a biased but partial representation, the motor system might integrate these biased signals to produce behavior, resulting in equal predictive power even without full redundancy in the memory representations themselves.

We agree with the reviewer that the integration of hemispheric errors may arise in the motor system. However, given the strong, above-chance decoding accuracy on ipsilateral trials (Fig. 2h), we find it unlikely that the neural-behavioral error correlations reflect solely biased, partial representations. Our interpretation as evidence for redundant hemispheres is indeed

based on a combination of the strong ipsilateral code (Fig. 2h) and the ipsilateral and contralateral neural-behavioral correlations (Fig. 3b).

We clarified this in the text:

“Therefore, in combination with the strong ipsilateral decoding (Fig. 2h), our analyses did not support the specialized model (Fig. 1a), but rather pointed toward a redundant architecture (Fig. 1b), with both hemispheres encoding stimuli and guiding behavior in the full visual field.” (lines 190-193)

Below are some minor points:

1. “BIC” should be defined on first use.

Done.

2. “Area 8Ar”: briefly note this is dorsolateral prefrontal for readers unfamiliar with macaque nomenclature cortex and note its role in spatial working memory.

Done.

3. The 30% threshold of reactivation strength appears arbitrary. Please clarify the rationale for choosing the threshold.

Indeed, this threshold was arbitrarily selected. We now show in Supplementary Fig. 8d that the results are similar for a range of thresholds.

4. Extended Data Fig. 7 misses label for panel C on the figure.

Added.

5. Missing statistics (p value) for Fig 6 simulation results.

Added.

6. Mixed use of PFC and prefrontal cortex. Please pick one for consistency.

Done.

<u>Reviewer #3 (Remarks on code availability):</u>

Code is prepared in clear structure and well documented. Cannot test the code since data is not available yet.

The data has been made available as: https://doi.org/10.1184/R1/31431415.

We have also added a link to a single session with instructions for use in the github repository https://github.com/comptelab/RedundantPrefrontalHemispheres, with all major analyses saved in files, such that the figures can be quickly assessed.

Bibliography

Almeida, R., Barbosa, J. and Compte, A. (2015) “Neural circuit basis of visuo-spatial working memory precision: a computational and behavioral study,” *Journal of Neurophysiology*, 114(3), pp. 1806–1818. Available at: https://doi.org/10.1152/jn.00362.2015.

Buschman, T.J. *et al.* (2011) “Neural substrates of cognitive capacity limitations,” *Proceedings of the National Academy of Sciences*, 108(27), pp. 11252–11255. Available at: https://doi.org/10.1073/pnas.1104666108.

Edin, F. *et al.* (2009) “Mechanism for top-down control of working memory capacity,” *Proceedings of the National Academy of Sciences*, 106(16), pp. 6802–6807. Available at: https://doi.org/10.1073/pnas.0901894106.

Kim, S. and Alais, D. (2021) “Individual differences in serial dependence manifest when sensory uncertainty is high,” *Vision Research*, 188, pp. 274–282. Available at: https://doi.org/10.1016/j.visres.2021.08.001.

Matsushima, A. and Tanaka, M. (2014) “Different Neuronal Computations of Spatial Working Memory for Multiple Locations within versus across Visual Hemifields,” *Journal of Neuroscience*, 34(16), pp. 5621–5626. Available at: https://doi.org/10.1523/JNEUROSCI.0295-14.2014.

McDonnell, M.E. *et al.* (2025) “Interactions across hemispheres in prefrontal cortex reflect global cognitive processing.” bioRxiv, p. 2025.06.12.659406. Available at: https://doi.org/10.1101/2025.06.12.659406.

Schneegans, S. and Bays, P.M. (2016) “No fixed item limit in visuospatial working memory,” *Cortex; a Journal Devoted to the Study of the Nervous System and Behavior*, 83, pp. 181–193. Available at: https://doi.org/10.1016/j.cortex.2016.07.021.

Schneegans, S. and Bays, P.M. (2018) “Drift in Neural Population Activity Causes Working Memory to Deteriorate Over Time,” *The Journal of Neuroscience: The Official Journal of the Society for Neuroscience*, 38(21), pp. 4859–4869. Available at: https://doi.org/10.1523/JNEUROSCI.3440-17.2018.

Stein, H. *et al.* (2020) “Reduced serial dependence suggests deficits in synaptic potentiation in anti-NMDAR encephalitis and schizophrenia,” *Nature Communications*, 11(1), p. 4250. Available at: https://doi.org/10.1038/s41467-020-18033-3.

Vallat, R. (2018) “Pingouin: statistics in Python,” *Journal of Open Source Software*, 3(31), p. 1026. Available at: https://doi.org/10.21105/joss.01026.

Wei, Z., Wang, X.-J. and Wang, D.-H. (2012) “From Distributed Resources to Limited Slots in Multiple-Item Working Memory: A Spiking Network Model with Normalization,” *Journal of Neuroscience*, 32(33), pp. 11228–11240. Available at: https://doi.org/10.1523/JNEUROSCI.0735-12.2012.

Wimmer, K. *et al.* (2014) “Bump attractor dynamics in prefrontal cortex explains behavioral precision in spatial working memory,” *Nature Neuroscience*, 17(3), pp. 431–439. Available at: https://doi.org/10.1038/nn.3645.

Zhang, H. and Alais, D. (2020) “Individual difference in serial dependence results from opposite influences of perceptual choices and motor responses,” *Journal of Vision*, 20(8), p. 2. Available at: https://doi.org/10.1167/jov.20.8.2.

Rebuttal letter

We thank the three reviewers for their positive feedback and appreciate their thorough reviews.

Reviewer #1:

I thank the authors for their thorough and careful response to my comments. They have addressed all of my concerns. I am impressed with the quality of the work and the rigor of the revised submission.

Thank you for your feedback and the previous review. We also believe it improved the manuscript substantially.

Reviewer #2:

The authors have answered all my questions.

Thank you for your previous feedback, which helped improve the manuscript substantially.

Reviewer #3:

Thank you for the careful and thorough revisions. The added results substantially strengthen the manuscript and transform the paper from presenting a speculative model to providing empirical validation of key claims. In figure 6g, a clever analysis on the existing monkey data demonstrates better behavioral precision with dual-hemisphere accuracy. In figure 7, analyses on human multi-item memory data directly supports the load-dependent switching between redundant and specialized storage. There are only a couple remaining minor points that may require additional clarification.

Thank you for your feedback and the previous review. We also believe it improved the manuscript substantially.

[Re: Point 3] The added discussion text addresses this well. One minor point: correlated noise from shared upstream visual areas (V4, LIP) could create spatially structured perturbations similar to direct callosal connections. This doesn't undermine your conclusions as both indicate interhemispheric coordination, but you might briefly acknowledge this alternative circuit mechanism.

Added in line 443:

“Uninstructed eye movements, the most plausible confound, did not alter the effect, though correlated noise from upstream areas could provide an alternative explanation.”

[Re: Point 4] Thanks for adding in the criteria for trial removal. Does the term "consecutive correct trials" mean that the n-th trial and the (n-1)-th trial should both be correct to be included in the analyses? Since this might influence the serial dependence results (e.g., under-estimating the effect due to reduced variance, or cleaning up the pattern with outliers removed), it would be helpful to include a short explanation for the choice to include only consecutive correct trials.

Yes, consecutive correct trials means that both the current trial and the immediately preceding trial had to be correct to be included in the analyses. Importantly, most excluded trials resulted from fixation breaks on the preceding trial, which could occur either before stimulus onset (rendering that trial irrelevant) or during the delay period (introducing uncontrolled variability in memory duration). In addition, restricting analyses to consecutive correct trials avoids ambiguity arising from incorrect responses on the preceding trial, where the previous choice and previous stimulus may diverge.

Added in lines 484-486:

"[...] and only analyzed consecutive correct trials, leaving a total of 18,184 trials (61.72% of all current rewarded trials). Consecutive correct trials were selected to avoid potential confounds arising from fixation breaks in the preceding trial, which accounted for the majority of excluded trials, and to prevent ambiguity introduced by incorrect responses on the preceding trial. "

[Re: Point 7] Figure 6g may actually provide additional evidence for your interpretation: if equal behavioral prediction reflected only motor integration of biased estimates, you wouldn't expect better precision when both hemispheres are accurate versus only one. This reinforces that redundancy is in memory representations themselves, not just motor readout. Worth briefly noting when discussing Fig. 6g.

Thank you for highlighting this. We agree that Fig. 6g further supports full hemispheric redundancy over the mentioned motor integration confound. However, the point of Fig. 6g is to specifically test a prediction of the attractor model in the data. We worry that if we relate back towards redundancy vs specialization, a point we addressed in Figures 2 and 3, we may dilute this prediction and the strength of the panel. We have therefore opted not to adapt the text.

I recommend accepting the paper with (optional) slight revision as discussed above.

Thank you for your reviews.
